# Molecular Assembly of a Durable HRP-AuNPs/PEDOT:BSA/Pt Biosensor with Detailed Characterizations

**DOI:** 10.3390/s18061823

**Published:** 2018-06-05

**Authors:** Fangcheng Xu, Shuaibin Ren, Jiansin Li, Xiang Bi, Yesong Gu

**Affiliations:** 1 Department of Chemical and Biochemical Engineering, Xiamen University, Xiamen 361005, China; fcxu@xmu.edu.cn (F.X.); rht0517@126.com (S.R.); 2 Department of Chemical and Materials Engineering, Tunghai University, Taichung 407, Taiwan; g05310019@thu.edu.tw (J.L.); g06310801@thu.edu.tw (X.B.)

**Keywords:** molecular assembling, poly(3,4-ethylenedioxythiophene), bovine serum albumin, gold nanoparticle, horseradish peroxidase biosensor

## Abstract

In this study, we provided the detailed characterizations of our recent HRP-AuNPs/PEDOT:BSA/Pt biosensor, constructed through a simple fabrication procedure with improved stability and good sensitivity. Raman and Fourier transform infrared spectroscopy and X-ray photoelectron spectroscopy not only confirmed the synthesis of conductive PEDOT where BSA was the template for the polymerization, but also provided further insights into the stable immobilization of AuNP on the PEDOT:BSA film. Scanning electron microscopy revealed that the attachment of AuNPs were stable under a high salt environment. The current technology demonstrates a feasible procedure to form a functional AuNPs/PEDOT:BSA film that has potential applications in the fabrication of various biosensors and electric devices.

## 1. Introduction

While great progress has been made in modern medicine, the development of various innovative diagnostic sensors is becoming extremely important for precision medicine. In comparison with traditional chemical and biochemical approaches that are often complicated and tiresome, researchers are working on constructing diagnostic biosensors with more convenient operation protocols [1]. A good biosensor is expected to possess good prospects in terms of convenience, cost-effectiveness, shelf-life, and biocompatibility [2]. Conductive polymers have attracted much attention in the design of various electrochemical sensors because conductive polymers have been found to dramatically eliminate the background interferences from bare electrodes, such as platinum, but provide better sensitivities to targets [3]. The PEDOT-coated biosensors have gained an obvious advantage in the quantification and qualification of DNA and other biomarkers [4,5,6]. However, the peeling of PEDOT film from Pt electrodes during multiple measurements has become the major drawback for further applications. We have attributed this phenomenon to the synthesis of short PEDOT fragments due to the lack of templates and the heterogeneous natures of the hydrophobicity of PEDOT film against the slight hydrophilicity of platinum. Recently, we have also demonstrated that the linearized proteins, such as bovine serum albumin (BSA), could be used as templates in the electrochemical synthesis of thermal stable PEDOT:BSA on the platinum (Pt) electrode [7].

Horseradish peroxidase (HRP) is a common enzyme that catalyzes the decomposition of H_2_O_2_, has well-known chemical properties and good stability, and is relatively cheap and commercially available [8]. HRP has not only been used in general biochemical analyses, but has also been applied in biosensor fabrications [3,9], polymer synthesis [10,11], and biofuel cells [12]. Our recent publication demonstrated that the thermal stable PEDOT:BSA film was able to immobilize the gold nanoparticles (AuNP) by means of a feasible molecular assembly procedure, and HRP was then captured to form the stable HRP-AuNPs/PEDOT:BSA/Pt electrode without using any chemical cross-linkers [7]. To further understand the mechanisms involved in automatic AuNP deposition on the PEDOT:PSS, we performed detailed characterization of the HRP-AuNPs/PEDOT:BSA/Pt biosensor using Fourier-transform infrared spectroscopy (FTIR), Raman spectroscopy, scanning electron microscope (SEM), and X-ray photoelectron spectroscopy (XPS). We also investigated the mechanism involved in the immobilization of HRP through the Au and SH interactions. Furthermore, we employed this biosensor to perform H_2_O_2_ measurements with differential pulse voltammetry (DPV).

## 2. Material and Methods

### 2.1. Chemicals

We purchased HRP, BSA, 3,4-ethylenedioxythiophene (EDOT), and nicotinamide adenine dinucleotide (NAD^+^) from Sigma-Aldrich Corp. (St. Louis, MO, USA), H_2_O_2_ (35%, *v*/*v*) and lithium perchlorate trihydrate (LiClO_4_) from Merck KGaA (Darmstadt, Germany). Only analytical grade chemicals were used in this study.

### 2.2. Instruments and Electrochemical Measurements

We employed a CHI-621B electrochemical analyzer (CHI instruments from Austin, TX, USA) to electrochemically synthesize PEDOT:BSA on the Pt electrode and performed all characterizations in a home-made miniature electrochemical cell. The modified PEDOT:BSA/Pt electrode (with a working area of 0.1912 cm^2^) served as the working electrode, while a bare Pt wire was the auxiliary electrode and an Ag/AgCl (in 3 M sodium chloride) electrode was the reference electrode.

Electrochemical synthesis of PEDOT:BSA film and the fabrication of HRP-AuNPs/PEDOT:BSA electrode were described in detail in our recent publication [7]. In this work, DPV was further employed to assess the function of this electrode by quantifying H_2_O_2_ in a 0.1 M PBS buffer with 50 μM of NAD^+^ and pH 6.2. Before adding H_2_O_2_, all buffers were purged with pure nitrogen for 30 min for deoxygenation. The profiles of DPV were reported in the potential ranger from −0.2 to 0.8 V with a scan rate of 50 mV/s. All experiments were finished in 0.1 M PBS (pH 6.2) under room temperature and a nitrogen atmosphere.

### 2.3. Characterizations of Electrode Surfaces

We used an ABT-150S SEM (TOPCON Corp., Tokyo, Japan) to visualize the surface morphologies of electrodes and a DA 8.3 FTIR spectrophotometer (Bomem Inc., Québec City, QC, Canada) to analyze the surface chemical compositions and modifications. A home-assembled confocal micro-Raman spectrometer (Tsinghua University in Taiwan) was employed to provide the Raman spectra from 200 cm^−1^ to 1700 cm^−1^. The X-ray Photoelectron Spectroscopy (XPS) with a VG ESCA Scientific Theta Probe was utilized for the analysis of the elemental compositions of the film. Monochromatized Al K_α_ radiation with an energy resolution of 0.8 eV was used to record the spectra. For all analyses, the energy calibration of the all species took the C1 s peak as a reference. After background correction, we conducted the deconvolution of the high-resolution spectra into Gaussian curves with XPS PEAK4.1 software.

## 3. Results and Discussion

### 3.1. Constructing HRP-AuNPs/PEDOT:BSA/Pt Electrode

**Figure 1:**
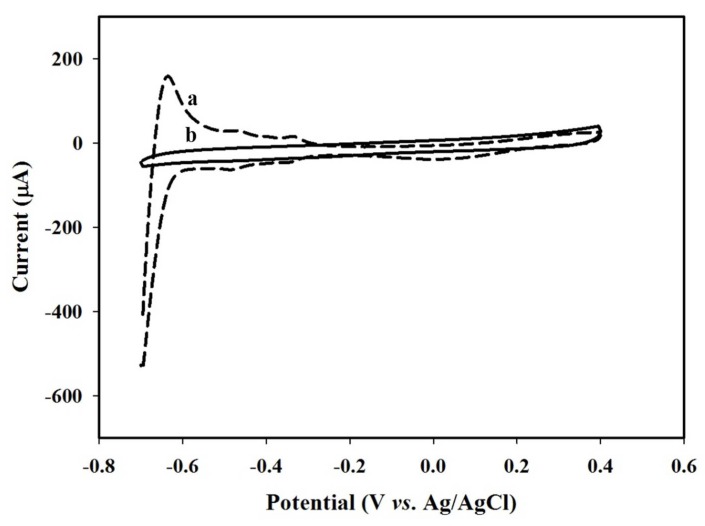
The profiles of cyclic voltammetry (CV) for (a) bare Pt, (b) modified PEDOT:BSA/Pt electrodes in 0.1 M PBS buffer with pH = 6.2 and a scan rate of 0.2 V/s.

In general, we often employ the cyclic voltammogram to evaluate the performance of HRP modified biosensors [3,7,9], which were normally carried out in the potential range of −0.6–0.8 V. Curve (a) in Figure 1 displays several redox peaks between −0.7 and −0.2 V for the bare Pt electrode, which implicates the adsorption-desorption of hydrogen following the electrolysis of H_2_O_2_. The overactive behavior of the bare Pt may result in the reduction of electrode selectivity [3]. By contrast, these redox peaks completely disappeared on the Pt electrode covered by an electrochemical synthesized PEDOT:BSA film (curve (b) in Figure 1), suggesting that the PEDOT:BSA was able to effectively avoid the unexpected electrochemical reactions on the surface of bare Pt. A similar phenomenon has also been reported for the PANI/Pt electrode [3].

**Figure 2:**
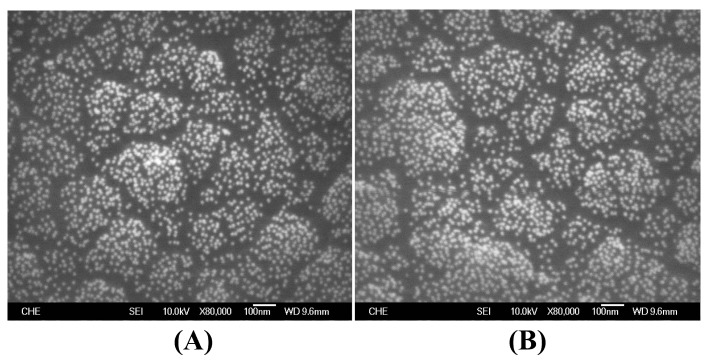
The images of SEM for the surface of AuNPs/PEDOT:BSA/Pt electrodes after being soaked in (**A**) distilled water, and (**B**) 3 M NaCl solution for two hours and air-dried.

Previously, we have often observed that the electrochemically synthesized PEDOT on the bare Pt electrode frequently suffered from severe degradation in sequential measurements. We then discovered that the BSA might take the same role as PSS in improving the durability of conductive PEDOT film [7]. In addition, we have noticed that AuNPs are difficult to immobilize on the PEDOT:PSS film, which is possibly due to the electric repulsion between AuNPs and exposed PSS (data not shown). Interestingly, our results demonstrated that the AuNP particles were able to be stably immobilized on the PEDOT:BSA films (Figure 2A). The AuNPs/PEDOT:BSA/Pt electrode was then able to capture the HRP molecules to contracture the HRP-AuNPs/PEDOT:BSA/Pt biosensor.

### 3.2. Electrochemical Measurements of H_2_O_2_

**Figure 3:**
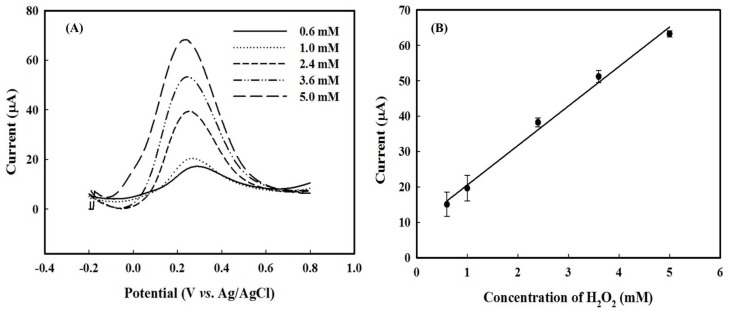
(**A**) The profile of differential pulse voltammetry (DPV) for electrode in response to H_2_O_2_; (**B**) The nice linear relationship between peak current and molar concentration of H_2_O_2_, where data were collected with a single electrode from triple-measurements for each concentration of H_2_O_2_.

We evaluated the performance of HRP-AuNPs/PEDOT:BSA/Pt electrode by measuring H_2_O_2_ in a PBS solution with cyclic voltammogram and obtained a very nice linear relationship between the cathodic peak current and the molar concentration of H_2_O_2_ [7]. Meanwhile, we also noticed that NAD^+^ contributes to the electron transport between HRP and the electrode, as well as the sensitivity of measurement, which reached 974.8 μA/(mM·cm^2^), both of which were better than others [13,14,15,16]. In this study, we presented the DPV profile of the measurement, in which oxidative peaks were observed near 0.3 V within the potential from −0.2 to 0.8 V and the peak current increased with the molar concentration of H_2_O_2_ with a nice linear correlation (Figure 3). Both CV and DPV measurements suggested that the HRP-AuNPs/PEDOT:BSA/Pt electrode possesses the good sensitivity and excellent stability.

### 3.3. FTIR Characterization of HRP-AuNPs/PEDOT:BSA/Pt Electrode

**Figure 4:**
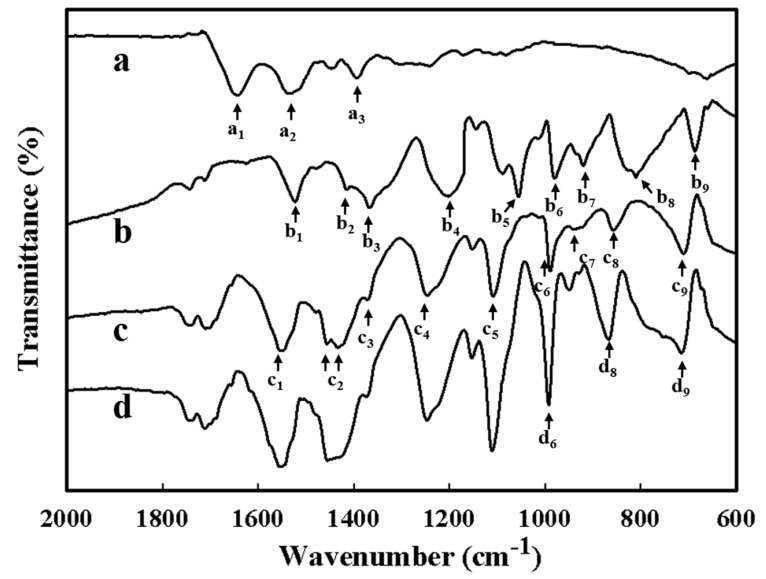
The FTIR spectra for (a) BSA, (b) PEDOT/Pt, (c) PEDOT:BSA/Pt, and (d) AuNPs/PEDOT:BSA/Pt.

To better understand the underlying chemical and physical alternations of the HRP-AuNPs/PEDOT:BSA/Pt electrode during preparation, we performed FTIR analysis for BSA, PEDOT/Pt, PEDOT:BSA/Pt, and AuNPs/PEDOT:BSA/Pt. Curve (a) in Figure 4 displays the typical spectrum of BSA, where the peaks at 1650 (a_1_) and 1530 (a_2_) cm^−1^ denote the C=O stretching vibration near the N–H bending and amide I as well as C–N stretching vibration near amide II, which are mainly associated with peptide backbone. The peak at 1390 (a_3_) cm^−1^ indicates the bending vibration of C–H in the aliphatic side chains of the amino acid residues [17,18].

On the other hand, curve (b) in Figure 4 presents the characteristic FTIR spectra of PEDOT [19], where peaks near 1532 (b_1_), and 1374 (b_3_) cm^−1^ are assigned to the asymmetric stretching vibrations of C=C and C–C in the thiophene ring, respectively. Peaks near 984 (b_6_), 816 (b_8_), and 692 (b_9_) cm^−1^ represent the stretching vibrations of C–S–C bond in thiophene ring. The peaks between 1062 (b_5_) and 1220 (b_4_) cm^−1^ are due to the bend vibration of C–O–C in ethylenedioxy ring, and the peak of 926 (b_7_) cm^−1^ is attributed to the deformation of ethylenedioxy ring. All the information has suggested the successful synthesis of the PEDOT through the electrochemical approach. 

In comparison with curves (a) and (b), curve (c) in Figure 4 demonstrates the combination pattern of spectra for BSA and PEDOT; however, the characteristic peaks for PEDOT all shift to the higher wavenumbers, which is an evident indication of intermolecular interactions between BSA and PEDOT. Among these, the peaks near 1062 (b_5_) and 816 (b_8_) cm^−1^ have shifted the most, to 1114 (c_5_) and 864 (c_8_) cm^−1^, respectively. Therefore, BSA interacted with PEDOT along its polymer chain and possibly served as the template during the electrochemically synthesis of PEDOT.

Nevertheless, curve (d) in Figure 4 suggests that the attachment of AuNPs does not cause any further shift for peaks identified for PEDOT, except that the peaks near 996 (d_6_), 880 (d_8_), and 730 (d_9_) cm^−1^ that are ascribed to C–S–C bond vibrations in the thiophene ring were greatly enhanced. These alternations imply that the AuNPs possibly made the direct approach to C–S–C to form the Au–S interaction.

### 3.4. Raman Characterization of HRP-AuNPs/PEDOT:BSA/Pt Electrode

**Figure 5:**
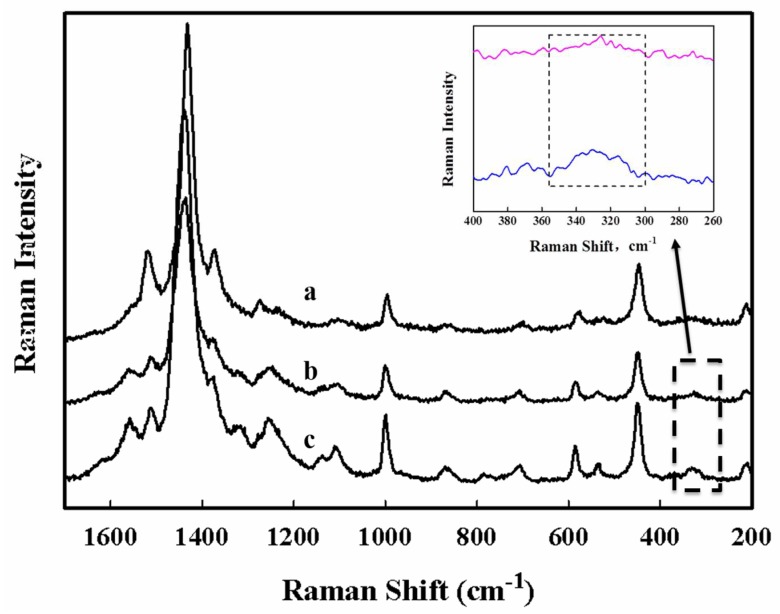
Raman spectra for electrodes, (a) PEDOT/Pt, (b) PEDOT:BSA/Pt, and (c) AuNPs/PEDOT:BSA/Pt. The inset indicates the expanded spectra between 260 and 400 cm^−1^ for curves (b) and (c).

FTIR and Raman are highly developed vibrational spectroscopies to explore the molecular motion and characterize chemical bonds of materials. Both techniques have their individual advantages and limitations; therefore, their combination may provide complementary information for the inspection of samples. The Raman spectra of three individual electrodes are exhibited in Figure 5.

Curve (a) indicates the Raman spectra of PEDOT, in which the major peaks that are strongly visible near 1518 cm^−1^ are assigned to the asymmetric vibrations of C=C in thiophene ring, whereas the peaks near 1432 and 1373 cm^−1^ are due to the symmetric stretching of C=C and stretching deformation of C–C. Peaks near 996 and 445 cm^−1^ are assigned to the oxyethylene ring deformation and the O–S–O bending vibration, respectively. A number of relatively weak peaks of PEDOT correspond to the C–C inter-ring stretching near 1275 and 1231cm^−1^, the deformations of C–O–C at 1104 cm^−1^, C–S–C near 698 cm^−1^, and oxyethylene near 574 cm^−1^ are present. The current spectra are quite similar to the previously reported Raman spectra for PEDOT [20,21,22].

In comparison with curve (a), curve (b) of the PEDOT:BSA/Pt electrode exhibits similar Raman spectra for PEDOT/Pt, with appearances of C=O vibration and S–S vibration near 1560 and 532 cm^−1^, respectively, indicating the entrapment of BSA in the PEDOT film. Meanwhile, we observe a similar blue shift of peaks, including peaks 1275 to 1313, 1231 to 1246, 996 to 1001, 698 to 706, and 574 to 585, which may further indicate the stable molecular interaction between BSA and PEDOT. Although PEDOT:BSA/Pt displays slightly weaker intensity of Raman spectra to that of PEDOT/Pt, the intensity for the stretching vibration of C–C inter-ring at 1246 cm^−1^ is enhanced, suggesting the longer chain of PEDOT probably synthesized in the presence of BSA.

In addition, curve (c) in Figure 5 displays the Raman spectra of AuNPs/PEDOT:BSA/Pt, where the intensity of the spectra is significantly enhanced with the attachment of AuNPs. There is no notable peak shift, but with the clear appearance of Au–S stretch near 330 cm^−1^, implicating the stable interaction between the AuNPs and the thiophene ring.

### 3.5. XPS Characterization of HRP-AuNPs/PEDOT:BSA/Pt Electrode

It has been deduced that electrostatic interactions contribute more to the formation of Au–S than the covalent interaction based on the theoretical calculation [23]. Accordingly, we explored the effect of a potentiostatic procedure applied to the electrode during the attachment of AuNPs, and found that more AuNPs were captured by applying a positive potential than a negative potential [7]. Therefore, electrostatic attraction did promote the Au-S interaction. On the other hand, we washed the electrode with a 3 M KCl solution or soaked the electrode in a 3 M KCl solution overnight, but there was no significant loss of AuNPs (Figure 2B). We had presumed that the established Au–S interaction would be much stronger than the general electrostatic interaction. Nevertheless, we were still not quite sure whether a covalent bond was formed between Au and S, since there was no clear evidence indicating the breakage of the S–C bond in the FTIR and Raman spectra.

**Figure 6:**
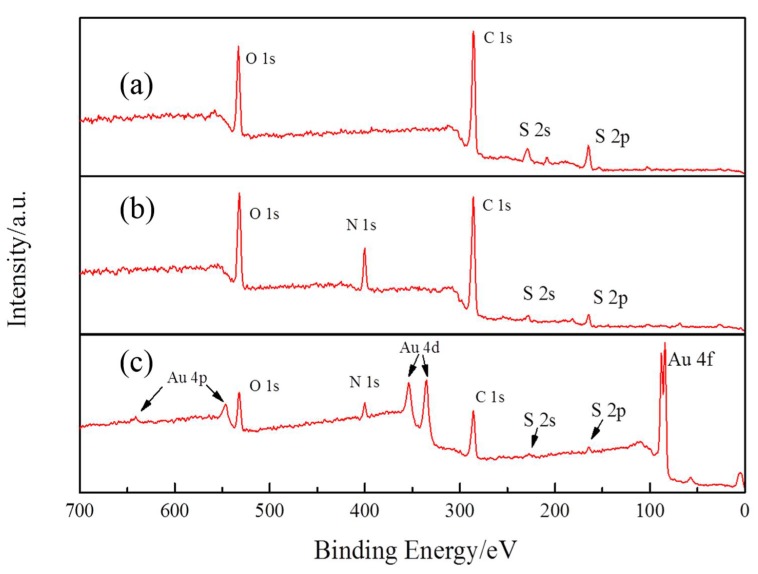
The overall XPS spectra of electrodes: (a) PEDOT/Pt, (b) PEDOT:BSA/Pt, and (c) AuNPs/PEDOT:BSA/Pt.

Based on the initial XPS spectra for PEDOT, PEDOT:BSA, and AuNP/PEDOT:BSA films in the binding energy range from 0 to 700 eV shown in Figure 6, we could clearly distinguish the differences among them, where Figure 6a exhibits the characteristic peaks of PEDOT with O 1s, C 1s, S 2s, and S 2p near 533, 285, 230, and 164 eV, respectively; Figure 6b displays an extra peak for N 1s near 400 eV, and the intensities of S 2s and S 2p were reduced, which was attributed to the exposure of BSA to the surface; and Figure 6c presents the appearance of Au spectra in corresponding to the doublet states of Au 4p, Au 4d, and Au 4f at 550 and 645, 340 and 354, 84, and 84.3 eV, respectively.

**Figure 7:**
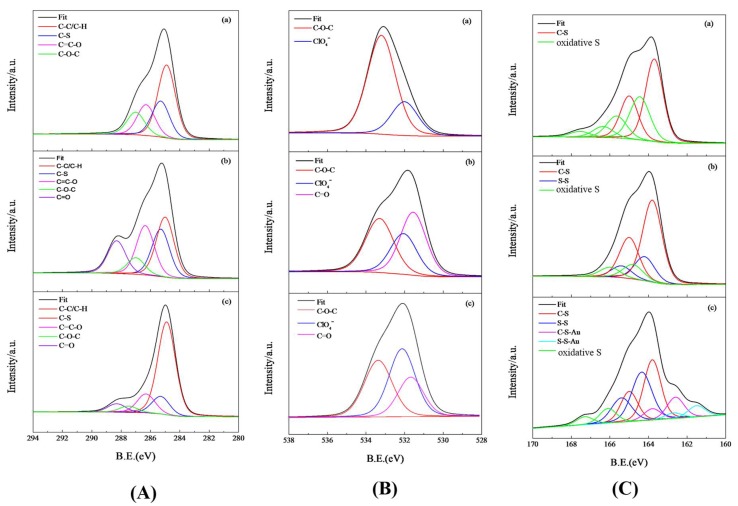
The fitting curves of deconvoluted spectra of XPS for (**A**) C 1s, (**B**) O 1s, and (**C**) S 1s, with (**a**) PEDOT/Pt, (**b**) PEDOT:BSA/Pt, and (**c**) AuNPs/PEDOT:BSA/Pt electrodes in each column.

To better understand the underlying information, individual spectra were characterized by curve fitting and deconvolution. The comparison of deconvoluted XPS C 1s spectra is shown in Figure 7A, where (a) displays the characteristic peaks of PEDOT, with C–H and C–C bonds near 284.9 eV, C–S bond near 285.3 eV, C=C–O bond near 286.3 eV, and C–O–C near 287 eV; (b) the new peak with a relatively high bonding energy near 288.3 eV results from the stable carboxyl group (–COOH) and peptide bond (–CONH–) in BSA, and the presence of BSA causes the reduction of the peak area ratios corresponding to C–H, C–C, and C–O–C bonds; and (c) the immobilization of AuNPs does not bring about any new peaks, but significantly enhances the intensity of the peaks for C–C and C–H.

By inspecting the deconvoluted XPS spectra of O 1s in Figure 7B, (a) we noticed the peak for C–O–C in PEDOT near 533.2 eV, whereas the peak near 532 eV was possibly due to the trace of LiClO_4_; (b) the presence of BSA brought about two new peaks, one was near 533.1 eV for C–O in –COOH and the other was near 531.5 eV for C=O in peptide bond, whereas the peak near 532 eV related to LiClO_4_ was not affected; and (c) after the attachment of AuNPs, we noticed a similar reduction of peaks corresponding to C–O–C and C=O to that found for C 1s.

Figure 7C demonstrates the deconvoluted spectra of S 2p, where (a) demonstrates the spin orbit splitting of S 2p in PEDOT, including S 2p_1/2_ near 165.0 eV and S 2p_3/2_ near 163.8 eV, with a binding energy difference of 1.2 eV where the ratio of peak areas was about 1:2. The green lines indicate about 42.6% of the S in PEDOT to be in oxidative state. Figure 7Cb represents the deconvoluted spectra of S 2p, including the similar spectra from PEDOT and the novel spectra from BSA. The blue lines indicate the spin orbit splitting of S 2p in the disulfide bonds of BSA with 2P _1/2_ near 165.4 eV and 2P _3/2_ near 164.2 eV. In comparison with Figure 7Ca, the shrunk areas under the green lines suggest the reduced oxidative state of S, which might implicate the more compact construct of PEDOT in the presence of BSA. In Figure 7Cc, due to the potential interaction with AuNPs, the bond energy of S 2p in C–S of PEDOT shifts to S 2p_1/2_ near 163.8 eV and S 2p_3/2_ near 162.6 eV, which might suggest that the formation of C–S–Au weakens the chemical bond of C–S. Although the bond energy of S 2p in the disulfide bonds of BSA is also reduced to S 2p_1/2_ near 162.7 eV and S 2p_3/2_ near 161.5 eV, the reduction of the peak area for S–S is less than that for C–S, which indicates that the interaction between Au and C–S is predominant.

**Figure 8:**
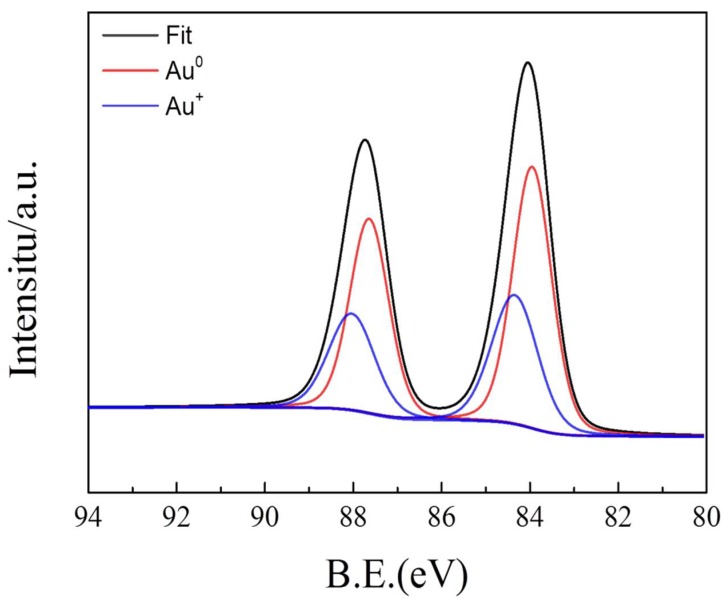
The fitting curves of deconvoluted XPS spectra for Au 4f on AuNPs/PEDOT:BSA/Pt electrode.

In addition, the XPS spectra of Au 4f for AuNP/PEDOT:BSA/Pt electrode are shown in Figure 8, which indicates Au 4f_7/2_ and Au 4f_5/2_ peaks with bond energy difference of 3/7 eV and a peak area ratio of 4:3. After deconvolution, the Au 4f_7/2_ has two spin-orbit splits of 84.0 eV and 84.3 eV, which implicate Au^0^ and Au^+1^, respectively. According to the results of FTIR and Raman, no clear breakage of C–S bonds in PEDOT was found after the immobilization of AuNPs, but notable reduction of XPS peak areas of S 2p_1/2_ and S 2p_3/2_ due to the Au immobilization; therefore, in the case of C–S–Au, Au is in its Au^0^ state and shares the pair of electrons in C–S. On the other hand, Au^+^ might be in the covalent interaction between S–S and Au. Interestingly, the peak area ratio for A^0^ and A^+^ is about 1.7:1 and closely corresponds to the peak area ratio of 1.9:1 for S 2p_3/2_ in C–S at 162.6 eV and in S–S at 161.5 eV.

## 4. Conclusions

To fabricate an enzyme-based biosensor, active enzymes are generally immobilized by either entrapment or chemical cross-linking, which often encounter the difficulties in maintaining high efficiency and good consistency. In this study, we proposed a feasible strategy to construct an HRP biosensor by modifying a platinum electrode with a conductive PEDOT:BSA film, which was able to capture AuNPs followed by immobilizing HRP through quick and simple soaking procedures. The biosensor displayed nice linear correlations between the electrochemical current and molar concentration of H_2_O_2_ with cyclic voltammetry (CV) and exhibited the main advantages of durability, sensitivity, and reliability [7]. Here, we further demonstrated its excellent performance with differential pulse voltammetry. Moreover, we explored the mechanisms involved in its construction, where FTIR and Raman spectroscopies revealed that BSA could serve as a template in the electrochemical polymerization of EDOT, but did not interrupted the polymerization of PEDOT (Figure 4 and Figure 5). Meanwhile, AuNPs possibly approached C–S–C directly to form the Au–S interaction. Furthermore, XPS analysis provided new evidence to demonstrate the stable interaction between Au and C–S in PEDOT, which contributed significantly to the stability of the HRP-AuNPs/PEDOT:BSA/Pt biosensor. The PEDOT:BSA/Pt electrode was confirmed to be cellular compatible (data not shown). The advantages of the current biosensor include feasible fabrication, durable function, acceptable sensitivity and selectivity, and biocompatibility. Furthermore, the constructed AuNPs/PEDOT:BSA conductive film should have potential applications in the development of novel electronic devices and biosensors.
